# Dr. Ephraim W. Gantt

**Published:** 1903-02

**Authors:** 


Dr. Ephraim W. Gantt, of Lockport, died in that city January 4, 1903, aged 71 years. He was born in Lockport, graduated in medicine at Columbia Medical College and practised for a few years in Illinois. Afterward he returned to Lockport and for many years was editor of the *Lockport Union*. He also served as a member of the Board of Education for several years.

